# How Can Design Features and Other Factors Affect the Indoor Air Quality in Inpatient Rooms? Check-Lists for the Design Phase, Daily Procedures and Maintenance Activities for Reducing the Air Concentrations of Chemical Pollution

**DOI:** 10.3390/ijerph17124280

**Published:** 2020-06-15

**Authors:** Marco Gola, Gaetano Settimo, Stefano Capolongo

**Affiliations:** 1 Architecture, Built environment and Construction engineering Dept, Politecnico di Milano, 20133 Milan, Italy; stefano.capolongo@polimi.it; 2 Environment and Health Dept, Istituto Superiore di Sanità, 00161 Rome, Italy; gaetano.settimo@iss.it

**Keywords:** Indoor Air Quality (IAQ), check-lists, chemical pollution, inpatient room, design features, best practices, management and design strategies, health prevention

## Abstract

Indoor Air Quality (IAQ) is one of main topics of Public Health on which international institutions and countries are taking action. With regards to healing architectures, several studies have reported data analysis and case studies to improve users’ health (patients, and medical and administrative staffs), but there are not enough regarding volatile organic compounds (VOCs). Regarding chemical pollution of indoor air, the Scientific Community has highlighted that there are several factors that affect the IAQ, in particular the design and management, and energetic efficiency, of inpatient wards. Several stakeholders, from the designers to the managers, are responsible for the indoor air in healing environments. Supported by analysis of the State of the Art and the main factors that influence the heterogeneous scenario of inpatient wards, the paper presents three check-lists, designed for supporting the stakeholders during the design phase, or for the daily procedures and maintenance activities, for pre-assessment of factors that affect chemical pollution, and for the definition of strategies to be applied. In fact, in such environments IAQ assumes a particular meaning and importance, both for the vulnerability of the patients and for the long time spent by the sanitary staff. The multidisciplinary approach emphasizes the continuous need for interdisciplinary knowledge and skills aimed at finding solutions able to protect users’ health status (including patients, workers and visitors), especially in the field of the indoor air issue.

## 1. Introduction: Indoor Air Quality in Hospital Settings

Several stakeholders are responsible for health promotion in indoor environments, starting with the decision makers and the designers during the design process, to the managers and the users who will manage, work, use and live in the spaces [1].

Referring to the factors that affect the Indoor Environmental Quality (IEQ), the Indoor Air Quality (IAQ) is an issue already discussed in the 1980s [2,3,4,5], although only in recent years has it become a strategic issue for all the countries [6], as one of the goals of the United Nations’ Sustainable Development.

Among the indoor environments, IAQ in “Architectures for Health” requires great attention for guaranteeing healthy indoor air and well-being, in particular for protecting patients and staff against Hospital Acquired Infections (HAIs) and occupational diseases [7,8]. In fact, it is well known that healthcare facilities are complex constructions, due to their dimensions, the high technologies for their functioning, the relevant presence of daily users, and their 24/7 operability [9].

Therefore, during the design process of hospital settings and their management, it is necessary to guarantee healthy spaces, starting with (a) the support of managers, medical and non-medical staff, maintainers and service providers, and (b) the design of hospital planners in relation to the layouts, the material selection, the rooms’ dimension, the engineering plants, etc., which can highly affect the performances of the facility and its processes [10,11]. In fact, as Joseph and Rashid sustained, the Scientific Community has shown that healthcare design may directly affect safety in hospitals, and it probably indirectly influences safety by triggering adverse events that cause harm to users (both patients and staff) [12]. Moreover, it may also affect the safety in “Architectures for Health” to counteract risk factors and harmful events. Moreover, referring to Reiling et al., cognitive psychologists have stated that environment has a significant impact on safety and human performances [13]. As several authors highlighted, humans do not always behave clumsily and they do not always make mistakes, but their performances are highly influenced when they live and/or work in badly-conceived and -designed healing spaces [14,15,16].

In general, the IAQ within hospitals is a complex and dynamic system, in which physical factors, and biological and chemical contaminants generated in indoor environments, as well as those in the outdoor air that come into the building via natural ventilation (i.e., through manual window opening) and, primarily, mechanical ventilation (i.e., Heating, Ventilation and Air Conditioning systems; HVAC) play a role. The quality of the management and maintenance of engineering plants [15], the cleaning and disinfection activities, and the quality assessment plans are also all relevant [15,17]. It is well-known that some concentrations of outdoor pollutants (i.e., VOCs, benzene, formaldehyde, etc.) are lower than indoor ones, because of the internal sources, including HVAC systems, building and finishing materials, hospital staff, and medical activities [18].

Regarding the chemical composition of indoor spaces, there may be threats to the air quality from a range of indoor and outdoor sources [19,20,21,22,23,24,25,26,27,28]. Indoor sources include building and finishing materials, furniture, cleaning activities and products, use of specific chemical agents and disinfectants, quality of air ventilation and health education management protocols [29]. Humans are also considered a cause of chemical emissions [30].

Currently in the European Legislation, VOC concentrations and IAQ for generic indoor environments are limited via acts and norms in some countries, as Settimo and D’Alessandro [1] investigated, and currently many suggestions are being made for low and medium care hospital settings, excluding surgery blocks, laboratories, etc., as Settimo [31] highlighted.

Nowadays, for assessing chemical pollution, it is necessary to monitor activity, but this is not always possible to set it up immediately, and data analysis requires a long time. Therefore, the aim of the paper is to give rise to three check-lists for reducing chemical pollution in inpatient wards, for supporting designers, hospital planners, medical directors and managers, to be applied during the design phase, and for the application of daily procedures and maintenance activities in the defining of strategies for healthy performances. The focuses of the check-lists are related, referring to the current trends in hospital design, and to facilities with mechanical ventilation and/or mixed systems (natural and mechanical ones).

## 2. The Hospital Wards and the Inpatient Room

### 2.1. General Requirements of the Inpatient Ward

The inpatient ward is a low–medium care environment, with the presence of hospital staff and users with weak health conditions. Typically, a traditional inpatient ward has about 28–32 beds with support services for medical activities and hospital staff, such as a nursing station, the head nurse office, several storages, a medical room, doctors’ offices, a kitchenette, a workers’ dining room and users’ restrooms [18].

In this functional area, one of the most important pathogen sources is related to the respiration of a potentially infectious user and related medical activities; most of the inpatients spend a lot of their time in beds, while medical staff spend a lot of their time in a ward, depending on the activities to be carried out [32,33,34,35].

In general, they may be exposed to a wide range of chemical pollutants emitted from several products, such as finishing materials, furniture and disinfectants, and regular control plans, etc. [36].

The configuration of the inpatient room, as well as the design and health-related trends, is affected by: (a) environmental factors, dimensional space and design features; (b) managerial factors, related to medical procedures, training and health education, maintenance and cleaning activities, etc.; (c) social factors, guaranteeing hospitable spaces for users [37]. With regard to this latter aspect, several impacts of environmental features on health and wellbeing in hospitals can be subdivided into indoor air and thermo-hygrometric parameters, environmental safety, proper and efficient ventilation, acoustic noise, finishing materials, furniture, lighting, external views, wayfinding, colors, ergonomics, accessibility, etc. [38,39,40,41].

### 2.2. Factors that Affect the Indoor Air Quality of Inpatient Rooms

Since of the scientific literature reports give very different outputs and results, and the resulting work from the analysis conducted by Gola [18,42] is divided into specific fields of interest, related to ventilation systems, construction and finishing materials, installations, components, etc. 

Starting from an analysis already conducted by Gola et al. [18], it is possible to classify the factors of indoor air into four macro-areas [37]:

- Outdoor and indoor microclimatic factors, which refer to the outdoor air, the solar exposure and indoor microclimatic parameters, such as temperature, relative humidity, air velocity, air change, etc. Although these factors can vary, they have a great influence on the IAQ and the performances of materials in the room and air fluxes.
- Management activities, which refer to the management and maintenance activities, ventilation systems, cleaning and disinfectant activities, control plan, etc., carried out in the room and in the functional units and/or in the entire facility. They can highly affect IAQ, but their emissions can be controlled through the applications of strategies, and at the same time they can be changed if their actions are dangerous for users.
- Design factors, which refer to all the components that characterize the inpatient room (spaces’ dimensions, furniture, finishing, etc.). In general, their emissions are constant, although in relation to their life, the emissions may decrease over the time.
- Human presence, which refers to the presence of users, their health status, and the medical activities carried out in the inpatient room. Their presence and application can vary, and therefore they can affect the indoor air in different modes.

As emerges from an analysis of the several factors, to overcome the criticisms related to the design of healing environments, interdisciplinary knowledge needs to be taken into account, including: the needs of users (hospital staff, patients, outpatients, visitors, etc.) related to their activities and therapies; nosocomial infections; applications of the technologies and ventilation systems (HVAC) needed to carry out the ordinary and specialist healthcare disciplines; risk analysis techniques for several functional units, including events caused by incorrect application of procedures; acceptable residual risk values and related sharing and management procedures [43].

In addition, designers, in collaboration with healthcare professionals, should design the healthcare settings according to the different uses, support the healthcare organization in identifying the most optimal solutions (for the daily technical, functional, economic and management aspects [44]), and elaborate and monitor the management and maintenance procedures of environments and systems. In addition, the managers should train the staff who will use or manage the spaces and systems, through the processing and updating of appropriate procedures, training courses and monitoring.

Exposure of hospital users to chemical pollutions is related to several aspects, related to product formulations, how and where the products are applied, the methods of use, the degree of aging of the various components, the type of maintenance activity, etc. [30]. As some outputs of the analysis highlighted, the research field should more deeply explore exposure of hospital users through monitoring and assessments of exposure concentrations. In fact, as Bessonneau et al. observed, data have to be confirmed in a multi-centric approach, and research efforts must be designed with regard to the possible health effects induced after inhalation exposure to a complex mixture of chemical compounds [30].

## 3. Strategies for Improving the IAQ in Inpatient Rooms

### 3.1. Definition of Check-Lists for Pre-Evaluating Performances of Inpatient Rooms

Starting from the application of several case studies and the evidence given by a detailed analysis of the scientific literature [18], IAQ is a complex and challenging issue both for designers and hospital managers, and also for the National Health System (NHS). To pursue efficient levels of IAQ, and to reduce chemical pollutants in hospital environments, these compounds must be considered during design, construction and operational phases, as well as biological and physical ones [45,46,47,48,49,50]. As several scholars have demonstrated, many illnesses due to IAQ occur because indoor air has not been adequately considered in different phases, from the design to the management [51].

As the World Health Organization (WHO) and several Institutions have already highlighted, for understanding the real performances and the IAQ, it is proper to do several monitoring activities for an adequate data analysis, and for the investigating of the factors that affect this issue [51,52]. Although monitoring activities are preferred because they are more reliable, there are not always possible to set it up immediately, and data analysis requires a long time [53].

For this reason, for supporting designers and medical directors, the research team produces check-lists for the pre-assessment of chemical pollution in inpatient rooms, during the design phase, daily procedures and maintenance activities.

These check-lists aim to be useful tools for the pre-assessment and prior verification of strategies and decisions to be applied, and, when these cannot be verified, to have an awareness and take action for avoiding possible risks for the hospital users regarding chemical pollution. They become a preliminary tool to define a future and broad protocol for supporting the healthcare organizations in the strategies to be applied.

These lists refer to several criteria, considered as factors that affect IAQ (defined on the basis of the results of the analysis of present norms) the State of the Art, several research projects and investigations, and the systematic reviews by scientific international researchers, supported by reports promoted by the research group Study for the Indoor Air Pollution of Istituto Superiore di Sanità (National Institute for Health) in Italy [54]. The list of the references is reported in detail in each document. Each check-list is composed of several criteria, as follows: (a) field of interest—this section highlights the area of interest of the topic; (b) criterion—this section defines the argument and the topic of the criteria; (c) definition—this section defines the contents of the criteria; (d) requirement—this section lists the requisite to be met; (e) answer—this section is to be filled in for checking the requisites with “yes” or “no” answers (“not applicable” in the case of intervention not performed and delivered).

#### 3.1.1. Check-List for the Design Phase

The aim of this check-list is to investigate and verify the design features of the room (dimensions, materials, etc.), its configuration and its installations. The check-list is particularly useful for designers and hospital planners during the design process.

The general scope of this list is to highlight possible deficiencies in the design phase, and to implement the project of the room through design strategies. This check-list is strongly related to the design of new healthcare facilities, or the renovation of an existing hospital. In this phase, the decision makers have a great responsibility, because it is possible to already define the management strategies, supported by the healthcare organization, for obtaining the best performances of the rooms, as well as of the cleaning and maintenance activities.

Starting from the answers, and in particular the negative ones, it will be possible to define strategies and solutions for improving the use and performances of the environmental unit for the medical activities.

In general, the check-list focuses on healthcare facilities with mechanical systems, responding to the current trends in hospital design.

As Table 1 shows, starting from the review of scientific reports, the check-list raises 18 topics, subdivided into 2 items related to room localization, 3 to microclimatic parameters, 4 to room configuration, 6 to ventilation system and 3 to materials and furniture.

The check-list aims to highlight some criticisms, related mainly to negative answers, for identifying strategies and solutions to be applied, where possible, because each hospital project is different.

The following list (Table 2) suggests strategies to be taken into consideration for healthy performances of the inpatient ward.

#### 3.1.2. Check-List for Daily Procedures

The check-list has the aim of investigating and verifying the daily procedures, the room occupancy and its performances. The aim of this list is to highlight possible deficiencies in medical and human activities, and to implement the procedures through management strategies.

Although design strategies require hard interventions, the check-list permits one to consider several factors and plan alternative actions related to the ventilation system (HVAC) and its efficiency, cleaning activities and detergents, a series of more specific activities of sanitation and disinfection, medical activities, and procedural adoption of prudential and adequate behavior.

Starting from the answers, and in particular the negative ones, it will be possible to define strategies and solutions for improving the use and performances of the environmental unit for the medical activities.

In general, the check-list focuses on healthcare facilities with mechanical systems.

As Table 3 shows, starting from the review of scientific reports, the check-list argues 20 topics, subdivided into 1 item related to room localization, 1 to room configuration, 3 to microclimatic parameters, 5 to ventilation system, 2 to medical activities, 6 to cleaning activities, 1 to human occupancy, and 1 to management activities.

The check-list aims to highlight some criticisms, related mainly to negative answers, for identifying strategies and solutions to be applied, where possible, because each healthcare project is different.

The following list (Table 4) suggests strategies to be taken into consideration for healthy performances of the inpatient ward during the daily procedures.

#### 3.2.3. Check-List for Maintenance Activities and Interventions

The check-list has the aim of investigating and verifying the condition of the room after the maintenance activity and/or intervention.

The aim of this list is to highlight possible deficiencies of the room and procedures before occupation of the room by users, and to apply management strategies.

The type of interventions can be different (extra-ordinary maintenance with furniture installation, pipe cleaning, etc.), i.e., hard or soft interventions in the room. In relation to complexity, the strategies may concern natural ventilation or HVAC systems and efficiency, medical activities and management procedures, the planning of cleaning and disinfection, activities’ assessment, and actions related to air monitoring for both chemical and biological agents.

Starting from the answers, and in particular the negative ones, it will be possible to define strategies and solutions for improving the use and performances of the environmental unit for medical activities.

In general, the check-list focuses on healthcare facilities with mechanical systems.

As Table 5 shows, starting from the review of the scientific reports, the check-list argues 13 topics, subdivided into 1 item related to room configuration, 5 to materials and furniture, 3 to maintenance activities, and 4 to cleaning activities.

The check-list aims to highlight some criticisms, related mainly to negative answers, for identifying strategies and solutions to be applied, where possible, because each activity requires different attentions.

The following list (Table 6) suggests strategies to be taken into consideration for healthy performances during maintenance activities and interventions.

## 4. Conclusions and Future Perspectives

It is clear that IAQ is a very broad topic, in which any variable can affect the performance of air in indoor environments. In fact, design and management strategies which may be adequate in relation to different procedures can decrease or increase the quality performances of the inpatient room, and the comfort of users [83,84].

Although current monitoring activities and data analyses are more reliable, the check-lists become useful tools for a pre-assessment and prior verification of strategies and decisions to be applied, and when they cannot be verified, they help one to have awareness and take action for avoiding possible risks to the users.

The multidisciplinary approach, supported by design, chemical and healthcare issues, demonstrates the need for interdisciplinary of knowledges and skills aimed at finding solutions able to protect the health status of users [85]. It is clear that the design and management decision-making, including the adequate choice of the construction site and hospital exposure [55,56], finishing materials and furniture [86,87,88], cleaning and maintenance activities [77,89], etc., which can affect the IAQ, must be carried out on the basis of scientific research and data. It is necessary that the decision-making team should be composed of several professionals, for guaranteeing a multidisciplinary and synergic design project.

The hospital system should be rethought, giving attention to the pollutant emissions, and providing buildings that, from the design phase up to the building’s realization and functioning, can maintain the safe conditions of the indoor environment [68,69,90]. In this regard, it is worth underlining that, for emission control, it is not enough only to define engineering plans and design solutions, but it is essential to consider all the factors and medical and maintenance procedures [89].

The paper aims at the elaboration of strategies for health promotion in hospital wards, from the chemical pollution point of view. In any case, it is possible to consider improvements, all the time, to the contents of the check-lists, thanks to the support of several research groups, the data analysis and monitoring activities supported by scientific methodologies, such as those of Gola et al. and Śmiełowska et al. [10,76], which are useful tools for supporting designers and managers, as well as all the users who can actively contribute to the reduction of health risks (which highlights the importance of users’ behavior and hygienic aspects) [31,91].

These activities help the healthcare organization and hospital staff, with a view to preventions and the planning of interventions into the activities and the use of sanitizing or disinfectant substances related to medical activities, and also helping them to be ready in the case of epidemics (i.e., SARS-CoV-2), particularly regarding the activity of disinfection [92].

Among the future perspectives, the new challenge is to investigate the correlations between the chemical and biological pollutants, and their effects on IAQ and the quality of the healthcare facility.

## Figures and Tables

**Table 1:** Check-list for the design phase.

Field of Interest	Criterion	Definition	Requisite	Answer
OUTDOOR ISSUES	HOSPITAL LOCALIZATION	The criterion investigates the neighborhood in which the hospital is localized, and in particular the presence of sources of pollution [55,56].	Is the hospital localized in an area affected by the presence of sources of pollution (high traffic, industries, etc.)?	□ yes □ no
			If yes, do the designers apply design and ventilation strategies for reducing outdoor pollution in indoor environments?	□ yes □ no
SOLAR EXPOSURE	ROOM EXPOSURE	The criterion investigates the solar exposition of the room, and technological solutions for reducing solar intake [57].	Is the room exposed to the south, east and west?	□ yes □ no
			If yes, does the façade host some (passive or active) shielding systems? Or do glasses have special treatments for heat reduction?	□ yes □ no
			Are there inside windows?	□ yes □ no
MICROCLIMATIC PARAMETERS	EXPECTED TEMPERATURE	The criterion analyzes the expected temperature values during the year [58].	Is the expected temperature, during the year, around 21–24 °C?	□ yes □ no
MICROCLIMATIC PARAMETERS	EXPECTED RELATIVE HUMIDITY	The criterion analyzes the expected relative humidity values during the year [58].	Is the expected relative humidity, during the year, around 45–60%?	□ yes □ no
MICROCLIMATIC PARAMETERS	EXPECTED AIR VELOCITY	The criterion analyzes the expected air velocity for heating and cooling [58].	Is the expected air velocity around 0.05 m/s a 0.25 m/s?	□ yes □ no
ROOM DIMENSION AND LAYOUT	ROOM DIMENSIONS	The criterion analyzes the dimensions of the room, referring to the studies by Escombe et al. [59].	Is the volume of the room greater than 31 m^3^?	□ yes □ no
ROOM DIMENSION AND LAYOUT	ANTEROOM IN THE INPATIENT ROOM	The criterion investigates the presence of an anteroom in the inpatient room. As the scientific literature highlighted, this area reduces the concentration of pollutants in the air from the adjacent corridors and the other rooms [60].	Is there an anteroom (with a height lower than the Patient Core) in the inpatient room?	□ yes □ no
ROOM DIMENSION AND LAYOUT	WINDOW OPENING	The criterion verifies if the windows can be opened for guaranteed air changes inside the healing space, or if there are other strategies for improving the indoor air [51].	Is it possible to open the windows?	□ yes □ no
			If yes, can the user open of the windows manually?	□ yes □ no
			If it is not possible to open them, are there some forced ventilation strategies for improving air changes?	□ yes □ no
ROOM DIMENSION AND LAYOUT	DOOR OPENING	The criterion verifies the door opening and door configuration, referring to studies by Kalliomäki et al. and Sehulster et al., [61,62,63,64].	Is there a sliding door or a hinged door with an undercut of about 1–1.5 cm?	□ yes □ no
VENTILATION SYSTEM	AIR HANDLING UNITS (AHUs)’ LOCALIZATION	The criterion investigates AHUs’ localization. Outdoor air is subject to different pollutant concentrations. It is preferable to have AHUs far from the vehicular traffic and affected by wind flows [15].	Are the AHUs localized in strategic areas of the hospital for extracting outdoor air with reduced amount of pollutants?	□ yes □ no
VENTILATION SYSTEM	FILTRATION EFFIENCY	The criterion verifies the air exchange rate expected for inpatient room, as stated by the American Society of Heating, Refrigerating and Air-Conditioning Engineers (ASHRAE) 170 [58] and UNI EN ISO 16890:2017 [65].	Does the ventilation system provide efficient filters (at least, respectively, 80%–85%, 99% and 75%) for air pollution reduction?	□ yes □ no
VENTILATION SYSTEM	EXCHANGE RATE	The criterion verifies the air exchange rate expected for inpatient room, as stated by ASHRAE 170 [58] and Decree of the President of the Italian Republic 14/01/1997 [66].	Does the inpatient room have an air exchange rate about 4–6 vol/h?	□ yes □ no
VENTILATION SYSTEM	MECHANICAL SYSTEM	The criterion analyzes mechanical systems in the hospital [67].	Does the air-handling system provide variable air volume?	□ yes □ no
VENTILATION SYSTEM	AIR FLOW AMONG THE ENVIRONMENTAL UNITS	The criterion verifies the presence of design strategies for guaranteeing air flow among the environmental units [67,68,69].	Is it ensured that the air flow from one space to another takes place through the slots in the walls, ceilings, floors and around the doors?	□ yes □ no
VENTILATION SYSTEM	EXPECTED PRESSURE	The criterion analyzes the expected pressure in the inpatient room [58].	Is the expected pressure of the inpatient room positive?	□ yes □ no
CONSTRUCTION MATERIALS	BUILDING STRUCTURE	The criterion considers the absorption level of VOC levels of materials (e.g., painting, adhesive and sealant, etc.). In fact, without careful monitoring of the installation phases, these materials can act as contaminant tanks, releasing long-term re-emissions into the indoor air [70].	Are the building structure and construction materials lower absorbent materials of VOC levels?	□ yes □ no
FINISHING MATERIALS and FURNITURE	MATERIALS’ PERFORMANCES	The criterion verifies if the designers have selected materials with minimal risks for health and healthiness of spaces [71].	Have the designers selected certificated materials? Or, do they analyze chemical composition and emissivity of the materials?	□ yes □ no
FINISHING MATERIALS and FURNITURE	MATERIALS’ PERFORMANCES	The criterion verifies if the designers have selected durable materials that facilitate cleaning activity [51].	Do the designers select durable materials and surfaces that facilitate cleaning and maintenance activities, with high performances?	□ yes □ no

**Table 2:** Strategies for healthy outcomes of the project.

Criterion	Criticism	Strategies
outdoor issues—hospital localization	If in the neighborhood there is the presence of sources of pollution,	high performances of ventilations systems are required, with regular cleaning of the filters, and it is requested to open the windows only in case of need.
solar exposure—room exposure,	If the room has solar exposure (especially south and west), and the façade and the windows lack passive or active shielding systems, or glasses lack special treatments for heat reduction,	it is suggested they introduce some internal curtains, which comply with the hygiene requirements for healing environments, to reduce as much as possible the accumulation of heat for avoiding the emission of the VOC concentrations by the materials and for maintaining adequate microclimatic factors.
Microclimatic parameters—expected temperature/relative humidity/air velocity.	Although they are theoretical data to be verified on site, if the project data do not respect these requirements,	it is requested they improve or reduce the temperature/relative humidity and/or air velocity through ventilation systems and/or natural ventilation systems for adequate air rate changes, and improving the microclimatic parameters.
room dimension and layout—room dimensions	If the volume is lower than 31 m^3^,	it is requested they punctually improve the air changes.
room dimension and layout—anteroom in the inpatient room	If there is not an anteroom in the inpatient room,	it is better to reduce the door opening, introducing a window in the door for supporting nurses’ room control from the corridor.
room dimension and layout—window opening	If it not is possible to open the windows manually,	it is suggested they acquire a smart system that guarantees natural ventilation.
room dimension and layout—door opening	If there is not a sliding door or a hinged door with an undercut of about 1–1.5 cm,	it is requested they be careful during the daily activities of door opening because of the increased air movement between the confined spaces. Major air intake systems can control the air movement.
ventilation system—AHU localization	If the AHUs are not localized in strategic areas of the hospital,	it is suggested they verify during the design phase possible other localizations, or define maintenance and control activities for the highest performances of the AHUs.
ventilation system—filtration efficiency	If the ventilation system does not provide efficient filters for air pollution reduction,	it is requested they improve the efficiency of the filters in the mechanical systems.
ventilation system—exchange rate	Although they are theoretical data to be verified on site, if the project data do not respect the air exchange rate,	it is requested they improve air exchange rate and/or improve natural ventilation strategies with window openings.
ventilation system—mechanical system	If the mechanical system does not provide variable air volume,	it is requested they introduce some strategies for variable air volume and/or improve natural ventilation strategies with window openings.
ventilation system—air flow among the environmental units	If it is not ensured that the air flow from one space to another takes place through the slots in the walls, ceilings, floors and around the doors,	it is requested they introduce some design strategies for guaranteeing air flow among the spaces.
ventilation system—expected pressure	If the expected pressure is not positive in the inpatient room,	any action should be taken in this consideration, starting from existing legislation that does not list any specific requirements. In any case, it is suggested they place air intake systems near the door and windows openings.
construction materials—building structure	If there are materials of high/medium VOC absorbent levels,	it is suggested they select materials with low VOC concentrations. Where this is not possible, it is necessary they guarantee adequate air rate change, especially in the first months after laying, and avoid high solar exposure.
finishing materials and furniture—materials’ performances	If the designers have not selected certificated materials (or if the healthcare organization has not imposed any certificated materials),	it is suggested they select certificated materials. Where this is not possible, it is necessary to guarantee adequate air rate change, especially in the first months after laying, and to avoid high solar exposure.
finishing materials and furniture—materials’ performances	If the designers have not selected durable materials and surfaces that facilitate cleaning and maintenance activities (or if the healthcare organization has imposed specific materials),	it is suggested they introduce adequate finishing materials, able to be cleaned with cleaning products with low VOC concentrations.

**Table 3:** Check-list for daily procedures.

Field of Interest	Criterion	Definition	Requisite	Answer
SITE CONSTRUCTIONS IN THE NEIGHBORHOOD	SITE CONSTRUCTION	The criterion verifies the presence of a site construction in the surroundings or inside the hospital, and possible influence on the hospital building [55,56].	Are there some site constructions inside of the hospital, the hospital borders or in the neighborhood of the hospital?	□ yes □ no
			If yes, are there some procedures in place to prevent renovation and remodeling activities from adversely affecting the building air supply?	□ yes □ no
ROOM DIMENSION AND LAYOUT	WINDOW OPENING	The criterion verifies if the windows can be opened for guaranteeing air changes inside the healing space [51].	Is it possible to open the windows?	□ yes □ no
			If it is not possible, are there some forced ventilation strategies for improving air changes?	□ yes □ no
MICROCLIMATIC PARAMETERS	TEMPERATURE	The criterion analyzes the temperature values during the year [58,72].	Is the temperature, during the year, around 21–24 °C?	□ yes □ no
MICROCLIMATIC PARAMETERS	RELATIVE HUMIDITY	The criterion analyzes the relative humidity values during the year [58,72].	Is the relative humidity, during the year, around 40–60%?	□ yes □ no
MICROCLIMATIC PARAMETERS	AIR VELOCITY	The criterion analyzes the air velocity for heating and cooling [58,72].	Is the air velocity, for heating, around 0.05 m/s to 0.20 m/s?	□ yes □ no
			Is the air velocity, for cooling, around 0.05 m/s to 0.25 m/s?	□ yes □ no
VENTILATION SYSTEM	CROWD INDEX	The criterion analyzes the crowd index [58,72,73].	Is the crowd index predicted for the environmental unit 0.08?	□ yes □ no
VENTILATION SYSTEM	AIR FLOW vs CROWD INDEX	The criterion analyzes the relationship between air flow and crowd index [58].	Depending on the crowding, is the air flow ≥ 11 liters/s per person?	□ yes □ no
VENTILATION SYSTEM	EXCHANGE RATE	The criterion verifies the air exchange rate for the inpatient room [58].	Does the inpatient room have an air exchange rate about 4–6 vol/h?	□ yes □ no
VENTILATION SYSTEM	AIR FLOW	The criterion investigates the air flow rate in the inpatient room [73].	Is the air flow rate between inlet and discharge maintained?	□ yes □ no
VENTILATION SYSTEM	PRESSURE	The criterion analyzes the pressure in the inpatient room, although legislation does not list any specific requirement.	Is the expected pressure of the inpatient room positive?	□ yes □ no
MEDICAL ACTIVITY	MEDICAL ACTIVITIES AND THERAPIES	The criterion verifies the presence of medical activities in the inpatient room that can affect the quality of the air, as Lu at al. and Hsu et al. observed [74,75].	Are there some medical treatments carried out in the inpatient room that could affect the quality of the air?	□ yes □ no
			If yes, are there some procedures for diluting pollution levels?	□ yes □ no
MEDICAL ACTIVITY	MEDICAL EQUIPMENT	Plastic infusion bags, blood bags, plastic film, injectors, etc., can emit low concentrations of pollutants [76]. The criterion investigates if this equipment is used, and if they can affect the breathing zone.	Are there some medical equipment that can emit chemical pollutants in the breathing zone?	□ yes □ no
			If yes, are they rather distant from breathing zone?	□ yes □ no
CLEANING ACTIVITY	NUMBER of CLEANING ACTIVITIES per DAY	The criterion verifies the frequency of cleaning activities for inpatient rooms [51].	Is the room cleaned, properly, at least twice a day?	□ yes □ no
CLEANING ACTIVITY	CLEANED AREAS	The criterion verifies the areas cleaned daily [72].	Is there floor, furniture and bathroom cleaning every day?	□ yes □ no
CLEANING ACTIVITY	FLOOR CLEANING PRODUCTS	The criterion verifies the contents of detergents for floor cleaning, and if they affect the performance of the floor [56].	Are the floors cleaned with detergents with chlorine-derivatives, quaternary ammonium salts or phenol?	□ yes □ no
			Are the detergents affecting the performance of floors?	□ yes □ no
CLEANING ACTIVITY	FURNITURE CLEANING PRODUCTS	The criterion verifies the contents of detergents for furniture cleaning, and if they affect the performance of furniture [77].	Is the furniture cleaned with detergents with quaternary ammonium salts, phenol or alcohols?	□ yes □ no
			Are detergents affecting the performance of furniture?	□ yes □ no
CLEANING ACTIVITY	BATHROOM CLEANING PRODUCTS	The criterion verifies the contents of detergents for bathroom cleaning [77].	Is the bathroom cleaned with detergents with chlorine-derivatives, quaternary ammonium salts or phenol?	□ yes □ no
CLEANING ACTIVITY	AIR CHANGE DURING and AFTER CLEANING ACTIVITY	The criterion verifies if windows are opened during and after cleaning activities [78].	Are windows opened during and after cleaning activities for 15–20 minutes?	□ yes □ no
			If it is not possible, are there some forced ventilation strategies for improving air changes?	□ yes □ no
HUMAN PRESENCE	SMOCKING ACTIVITY	The criterion investigates the presence of warnings and design strategies applied for avoiding smoking in the healing environments. It is well-known that smoking can highly affect the indoor values [18,79].	Are there any smoking restrictions in the inpatient room or in the inpatient ward, or outside?	□ yes □ no
MAINTENANCE ACTIVITY	SAMPLING ACTIVITY	The criterion verifies if there are some monitoring activities for IAQ in the inpatient ward [72].	Are there some sampling activities for registering the indoor air in the inpatient ward?	□ yes □ no

**Table 4:** Strategies for healthy outcomes in daily procedures.

Criterion	Criticism	Strategies
site constructions in the neighborhood—site construction	If there are some site constructions inside of the hospital, the hospital borders or in the neighborhood of the hospital, and procedures have not been taken to prevent renovation and remodeling activities from adversely affecting the building air supply	it is suggested they define healthcare strategies for good outcomes of medical practices. In any case, good performance of ventilations systems is required, with regular cleaning of the filters, and it is requested they open the windows only in the case of need, for avoiding dust concentrations.
room dimension and layout—window opening	If it not is possible to open the windows	it is suggested they improve the air rate changes.
microclimatic parameters—temperature/relative humidity/air velocity.	If the parameters are not respected	it is requested they improve or reduce the temperature/relative humidity and/or air velocity through ventilation systems and/or natural ventilation systems, for adequate air rate changes and improving the microclimatic parameters.
ventilation system—crowd index	If the crowd index predicted for the environmental unit is not respected	it is suggested they improve the air rate changes.
ventilation system—exchange rate	If the air exchange rate is not respected	it is requested they improve air exchange rate and/or improve natural ventilation strategies with window openings.
ventilation system—air flow	If the air flow rate between inlet and discharge is not maintained,	it is suggested they improve the adequate air rate changes
ventilation system—pressure	If the pressure is negative,	any action should be taken into consideration, starting from existing legislation that does not list any specific requirement. In any case, it is suggested they have air intake systems near the door and windows openings.
medical activity—medical activities and therapies	If there are some medical treatments that can affect the quality of the air	it is requested they dilute the pollution levels, or, in general, improve the air rate changes.
medical activity—medical equipment	If there is some medical equipment that can emit chemical pollutants in the breathing zone of the user,	it is requested they maintain an adequate distance from the breathing zone (at least 1 mt).
cleaning activity—number of cleaning activities per day/cleaned areas/floor cleaning products/furniture cleaning products/bathroom cleaning products	In general, it is expected that at least twice a day the healing spaces are cleaned. If they are not,	it is requested they do the cleaning activities at least twice a day.
	In general, it is expected that cleaning products are differentiated. If they are not,	it is expected that the cleaning products are adequate for each surface, responding to the performance of the material.
cleaning activity—air change during and after cleaning activity	If the windows are not opened enough during and after cleaning activities,	it is requested they take into consideration this best practice, especially if the cleaning products have VOC concentrations. The window can be opened manually or with smart automatization system.
	If it is not possible to open the windows,	they is expected to improve the air rate changes.
human presence—smoking activity	If smoking restrictions in the inpatient room and in the inpatient ward, as well as outside, are lacking,	smoking restriction in healing settings are suggested, and/or the introduction of some sensors for monitoring the performance of the quality of the air.
maintenance activity—sampling activity	If the healthcare organization does not consider the introduction of sampling activities for registering the indoor air in the inpatient ward,	it is suggested they introduce some mobile sensors (which can also be used in different healthcare environments) for monitoring the quality of the air.

**Table 5:** Check-list for maintenance activities and interventions.

Field of Interest	Criterion	Definition	Requisite	Answer
ROOM DIMENSION AND LAYOUT	TYPE OF INTERVENTION	The criterion investigates the typology of intervention applied in the room. In relation to the activities carried out, it is possible to develop several considerations related to management strategies to be applied [55,56].	Do the interventions involve hard modifications of the room?	□ yes □ no
			If not, do the interventions involve punctual modifications of the room?	□ yes □ no
			If not, do the interventions involve maintenance activities on the installations and/or ventilation pipes of the room and/or inpatient ward?	□ yes □ no
CONSTRUCTION MATERIALS	MATERIALS’ CERTIFICATIONS	The criterion verifies if the designers have selected materials with minimal risks for health and healthy spaces [71].	Have the designers selected certificated materials?	□ yes □ no □ not applicable
			If not, do they analyze chemical composition and emissivity of materials, selecting the healthier ones?	□ yes □ no □ not applicable
CONSTRUCTION MATERIALS	MATERIALS’ PERFORMANCES	The criterion verifies if the designers have selected durable materials that facilitate cleaning activity [80].	Do the designers select durable materials that facilitate cleaning and maintenance activities?	□ yes □ no □ not applicable
FINISHING MATERIALS	MATERIALS LAYING	The criterion investigates the completeness of the work performed and the performances of materials [80].	Have the materials been laid correctly?	□ yes □ no □ not applicable
FINISHING MATERIALS	MATERIALS TREATMENT	The criterion investigates the treatments adopted for improving materials’ performances (linoleum, wood, etc.) [80].	Have finishing materials been treated with chemical agents to improve their performance?	□ yes □ no □ not applicable
FURNITURE	FURNITURE INSTALLATION	The criterion investigates the completeness of furniture installation, without any damages to the material that can affect the performance of the materials [18,72].	Has the furniture been installed correctly?	□ yes □ no □ not applicable
MAINTENANCE ACTIVITY	VENTILATION SYSTEM	Although typical ventilation systems are not affected by chemical pollution, the risk can be caused by the introduction of inadequate cleaning products. The criterion evaluates the products used for cleaning ventilation pipes. For this reason, the number of cleaning activities is not compulsory, even if inadequate cleaning products do not affect the indoor air [15].	Has the ventilation system been cleaned properly, with the use of adequate cleaning agents and disinfectants?	□ yes □ no □ not applicable
MAINTENANCE ACTIVITY	ACTIONS AFTER INSTALLATION	The criterion verifies if the managers of the hospital have guaranteed adequate ventilation in the room for emissions’ reduction after the installation/intervention [81].	Have the managers guaranteed the adequate ventilation for a minimum of 72 h after products’ installation/intervention?	□ yes □ no
MAINTENANCE ACTIVITY	ROOM OCCUPANCY	The criterion investigates the regular occupation of the room [81]. The intervention/installation in the first months can cause high emissions of pollution (whose peaks are registered in the first days after installation), so it is essential to ensure a ventilation rate greater than normal conditions in order to eliminate contaminants from the indoor settings [81].	Is the inpatient room regularly occupied by users, after a few days from the interventions?	□ yes □ no
			Is there a ventilation rate greater than normal conditions, in order to dilute contaminants from the room?	□ yes □ no
CLEANING ACTIVITY	ROOM CLEANING AFTER INTERVENTION	After maintenance activities and interventions, the hygienic conditions of the room are not adequate for users’ health status. For this reason, the criterion verifies if the room has been cleaned properly [77,81].	Has the room been cleaned properly after the intervention?	□ yes □ no □ not applicable
CLEANING ACTIVITY	ROOM CLEANING AFTER INTERVENTION	The criterion investigates the composition of detergents, verifying the consistency of the cleaning products on finishing and furniture [51].	Do the cleaning products comply with finishing materials?	□ yes □ no □ not applicable
CLEANING ACTIVITY	AIR CHANGE AFTER (EXTRA-ORDINARY) CLEANING ACTIVITY	The criterion verifies if an adequate air change has been guaranteed in the room after the (extra-ordinary) cleaning activity [80,82].	Have the cleaners opened the windows after (extra-ordinary) cleaning activities?	□ yes □ no □ not applicable
			If it is not possible to open the windows, have some forced ventilation strategies been applied for improving air changes?	□ yes □ no □ not applicable
CLEANING ACTIVITY	WINDOWS WASHING	The criterion verifies the detergents used for (extra-ordinary) windows washing in the inpatient rooms [51].	Are the windows washed with detergents with alcohols or phenol?	□ yes □ no □ not applicable

**Table 6:** Strategies for healthy outcomes for maintenance activities and interventions.

Criterion	Criticism	Strategies
room dimension and layout—type of intervention	In relation to the hard or soft maintenance activities to be done,	it is requested they pay attention to the healthcare flows and define all the useful actions for reducing the potential risks for the users.
construction materials—materials’ certifications	If the designers have not selected certificated materials (or the healthcare organization has not imposed any certificated materials),	it is suggested they select certificated materials. Where it is not possible, it is necessary to guarantee adequate air rate change, especially in the first months after laying, and to avoid high solar exposure.
construction materials—materials’ performances/materials laying/materials treatment	If the designers have not selected durable materials and surfaces that facilitate cleaning and maintenance activities (or the healthcare organization has not imposed specific materials),	it is suggested they introduce adequate finishing materials, able to be cleaned with cleaning products with low VOC concentrations. Where it is not possible, it is necessary to guarantee adequate air rate change, especially in the first months after laying, and to avoid high solar exposure.
furniture—furniture installation	If the furniture has not been installed correctly,	it is requested they guarantee adequate air rate change, especially in the first months after furniture installation, and avoid high solar exposure.
ventilation system—exchange rate	If it is not respect the air exchange rate,	it is requested they improve air exchange rate and/or improve natural ventilation strategies with window openings.
ventilation system—air flow	If the air flow rate between inlet and discharge is not maintained,	it is suggested they improve the adequate air rate changes.
ventilation system—pressure	If the pressure is negative,	any action should be taken into consideration, starting from existing legislation that does not list any specific requirement. In any case, it is suggested they have air intake systems near the door and window openings.
maintenance activity—ventilation system/actions after installation	If the ventilation system has not been cleaned properly,	it is suggested they use proper detergents, and guarantee adequate ventilation for a minimum of 72 h after products’ installation/intervention.
maintenance activity—room occupancy	If the room needs to regularly occupied by users a few days after the interventions,	it is requested they guarantee adequate air rate change, especially in the first days after the intervention.
cleaning activity—room cleaning after intervention/air change after (extra-ordinary) cleaning activity	In general, it is expected that adequate cleaning activities have been done correctly and cleaning products have been adequate for each surface. If it is not,	it is expected that the cleaning products are adequate for each surface, responding to the performance of the material.
	If an adequate air change has not been guaranteed in the room after the (extra-ordinary) cleaning activity,	it is requested they improve the natural or mechanical air rate changes before the use of the users.
cleaning activity—windows washing	If the windows are not washed with adequate detergents,	it is requested they use adequate products for the surface, responding to the performance of the material. In any case, adequate air change can reduce the VOC emissions.

## Abbreviations

AHUs	Air Handling Units
ASHRAE	American Society of Heating, Refrigerating and Air-Conditioning Engineers
HAIs	Hospital Acquired Infections
HVAC	Heating, Ventilation, and Air Conditioning systems
IAQ	Indoor Air Quality
IEQ	Indoor Environmental Quality
NHS	National Health System
SARS-CoV-2	Severe acute respiratory syndrome Coronavirus 2
VOCs	Volatile Organic Compounds
WHO	World Health Organization
